# Dietary Soybean Lecithin Improves Growth, Immunity, Antioxidant Capability and Intestinal Barrier Functions in Largemouth Bass *Micropterus salmoides* Juveniles

**DOI:** 10.3390/metabo13040512

**Published:** 2023-04-02

**Authors:** Jiaojiao Wu, Wenxue Yang, Rui Song, Zhe Li, Xiaowei Jia, Hao Zhang, Penghui Zhang, Xinyu Xue, Shenghui Li, Yuanyuan Xie, Rongfei Zhang, Jinyun Ye, Zhijin Zhou, Chenglong Wu

**Affiliations:** 1 National-Local Joint Engineering Laboratory of Aquatic Animal Genetic Breeding and Nutrition (Zhejiang), Huzhou University, 759 East 2nd Road, Huzhou 313000, China; 2 Zhejiang Provincial Key Laboratory of Aquatic Resources Conservation and Development, College of Life Science, Huzhou University, 759 East 2nd Road, Huzhou 313000, China; 3 Huzhou Agricultural Science and Technology Development Center, 768 Luwang Road, Huzhou 313000, China

**Keywords:** *Micropterus salmoides*, soybean lecithin, haematology, antioxidant capabilities, immune responses

## Abstract

This study evaluated the effects of dietary soybean lecithin (SBL) on the growth, haematological indices, immunities, antioxidant capabilities, and inflammatory and intestinal barrier functions because little information of dietary SBL could be obtained in juvenile largemouth bass (*Micropterus salmoides*). The fish were fed identical diets except for SBL added at 0, 2, 4 and 8%. It was found that 4 and 8% SBL significantly increased fish weight gain and daily growth rate (*p* < 0.05), while 4% SBL was optimal for enhancing RBC, HGB, PLT, MCV, MCH, WBC and MON in blood, and ALB and ALP in serum (*p* < 0.05). SBL (4%) also significantly elevated the antioxidant enzymes activities of T-SOD, CAT, GR, GPx, GST and T-AOC and GSH contents; increased mRNA transcription levels of Nrf2, Cu/Zn-SOD, CAT, GR, GST3 and GPx3; and decreased MDA contents. Keap1a and Keap1b levels were markedly down-regulated (*p* < 0.05). SBL (4%) significantly enhanced levels of the immune factors (ACP, LZM and C3) and the mRNA expression levels of innate immune-related genes (C3, C4, CFD, HEPC and MHC-I) compared with the control groups (0%) (*p* < 0.05). SBL (4%) significantly increased IgM and T-NOS in the intestine (*p* < 0.05) and significantly decreased levels of TNF-α, IL-8, IL-1β and IFN-γ and increased TGF-β1 at both transcription and protein levels in the liver and intestine (*p* < 0.05). The mRNA expression levels of MAPK13, MAPK14 and NF-κB P65 were significantly decreased in the intestine in the 4% SBL groups (*p* < 0.05). Histological sections also demonstrated that 4% SBL protected intestinal morphological structures compared with controls. This included increased intestinal villus height and muscular thickness (*p* < 0.05). Furthermore, the mRNA expression levels of the intestinal epithelial cell tight junction proteins (TJs) (ZO-1, claudin-3, claudin-4, claudin-5, claudin-23 and claudin-34) and mucin-5AC were significantly up-regulated in the 4% SBL groups compared with the controls (*p* < 0.05). In conclusion, these results suggested that 4% dietary SBL could not only improve growth, haematological indices, antioxidant capabilities, immune responses and intestinal functions, but also alleviate inflammatory responses, thereby providing reference information for the feed formulations in cultured largemouth bass.

## 1. Introduction

As by-product of soybean oil production, soybean lecithin (SBL) plays important nutritional roles for its high content of phosphatidyl choline, phosphatidyl ethanolamine, phosphatidyl inositol, phospholipids (PL) contents and trace amounts of glycolipids and triglycerides [1]. SBL could function as a food additive and improve growth, bolster immunity and increase antistress abilities in humans [2] and in terrestrial animals (cattle, chickens and turkeys) [1,3,4]. However, the limited biosynthetic capacity of PL could not meet the requirements for the normal growth and development in larval and juvenile aquatic animals [5]. Therefore, exogenous SBL was added into aquatic animal feeds as a primary non-protein raw material owing its ready availability [6,7,8]. Moreover, it has been widely used in many cultured fish species including golden mahseer (*Tor putitora*) [6], gilthead seabream (*Sparus aurata*) [8], Caspian brown trout (*Salmo trutta caspius*) [9], common carp (*Cyprinus carpio*) [10], stellate sturgeon (*Acipenser stellatus*) [11], rock bream (*Oplegnathus fasciatus*) [12] and channel catfish (*Ictalurus punctatus*) [13].

Many studies have found dietary SBL could not only increase growth, but also enhance health status, including innate immune and antioxidant responses in animals [9,11]. It is well-known that animal immune status could be monitored using haematological and antioxidant parameters [9,14]. Higher values of haemoglobin (HGB), red blood cells (RBC) and white blood cells (WBC) induced by adequate nutrients and probiotics could reflect better immunity and oxygen-carrying capacity via the haematopoietic system in many fish species [15,16]. Meanwhile, antioxidant capabilities can reflect the animal responses to reactive oxygen species (ROS) produced in normal metabolic and immune defence processes, while excessive ROS is deleterious, resulting in oxidative damage and impairment of physiological functions [17]. These antioxidant enzymes activities could be regulated by the Nrf2/Keap1 signalling pathway to maintain redox homeostasis [18], which is also closely linked to fish health status and innate immunity [19]. However, little information could be obtained about the relationships among the antioxidant capabilities, Nrf2/Keap1 pathway and dietary SBL in fish.

Encounters with nutrient-deficient, pathogens or environmental toxins could induce inflammatory processes and ROS products, which are the primary immune defence lines for protecting animals [20]. In particular, the p38 MAPK/NF-κB signalling pathway is responsible for pro- and anti-inflammatory cytokines production [21], while excessive immune stimulation could cause typical ‘cytokine storm’ mediated by pro-inflammatory mediators, including interleukin-1β (IL-1β), interferon-γ (IFN-γ) and tumour necrosis factor-α (TNF-α), etc. [22,23]. Many studies have demonstrated that overproduction of inflammatory cytokines could result in tissue damage and inappropriate physiological responses [24]. However, these processes are normally counteracted by increasing contents of anti-inflammatory cytokines, such as interleukin 10 (IL-10) and transforming growth factor β 1 (TGF-β1), that participate in tissue repair processes [5,24,25]. Therefore, it is essential to alleviate inflammation and improve immunity through regulating the balance between pro- and anti-inflammatory cytokine productions mediated by adequate nutrients and/or additives in animals [25].

In addition, the intestinal barrier function of fish was closely related to its health status. Intestinal barrier functions and integrity were associated with elevated levels of the epithelial cell tight junction proteins (TJs) occludin, zonula occludens and claudin [5]. These cells also maintain the mucus barrier using mucin-linked carbohydrates that form a protective gel that directly interacts with the intestinal microbiota [26]. These barrier protective functions could be altered by balanced nutrition in largemouth bass (*Micropterus salmoides*) [27], grass carp (*Ctenopharyngodon idella*) [28], jian carp (*Cyprinus carpio* var. Jian) [29] and Cobia (*Rachycentron canadum*) [30]. In contrast, the effects of dietary SBL on intestinal barrier functions were little described in aquatic animals.

Largemouth bass (*Micropterus salmoides*) is a typical carnivorous freshwater fish that is widely cultivated due to its rapid growth, strong adaptability and better meat quality. This species is one of the most important commercial freshwater species cultivated in China and its production reached 0.7 million tons in 2021 [31]. Preliminary studies using dietary phospholipids and intestinal health indices for largemouth bass larvae have been conducted but adults or juveniles were not included [5]. In the current study, we evaluated how SBL affects *M. salmoides* both at the transcriptional and protein levels, and further explored the mechanism of dietary SBL on the intestinal barrier functions and immune health using haematological indices and measures of inflammation and redox stress as well as intestinal tight junction (TJ) and mucin protein expression. These data provide a reference to optimize compound feed formulations for largemouth bass.

## 2. Materials and Methods

### 2.1. Experimental Diets

Four diet formulations were produced that included defatted fish meal (1.8% lipid), casein and gelatine as protein sources, and soybean oil and SBL as lipid sources. The ingredients were mechanically forced through 60-mesh screens for mixing. The 4 formulations provided identical levels of nitrogen, energy sources and lipids but contained graded levels of SBL and 0, 2, 4 and 8% substituted *w/w* for soybean oil, respectively (Table 1). SBL contained 196, 132 and 146 mg/g phosphatidylcholine, phosphatidylethanolamine and phosphatidylinositol, respectively, and was analysed using high-performance liquid chromatography with evaporative light scattering detector (HPLCELSD). The components were processed into 2.5 mm diameter pellets using a commercial F-26 twin-screw extruder (South China University of Technology Machinery Factory, Guangzhou, China). The pellets were air-dried at 35 °C and stored at −20 °C. The proximate composition of these four experimental diets were determined according to the methods described by Jia et al. [17].

### 2.2. Experimental Fish and Feeding Trial

*M. salmoides* juveniles were obtained from Deqing Longshenli Biotech Co., Ltd., (Huzhou, China) and cultured in the recirculating aquaculture system of Huzhou University. Before the start of the normal feeding experiments, the fish was disinfected and placed in 500 L tanks for one week and domesticated using commercial feed (Deqing Longshenli). Disease- and injury-free juveniles (360) weighing 9.33 ± 0.06 g were selected and distributed randomly into 12 tanks (90 fish per experimental diet). The fish were fed twice a day at 08:00 and 17:00 for 8 weeks. Uneaten feed was removed by siphoning every 2 days, and the same amount of fresh water was returned to the tanks. Water temperature was maintained at 26–29 °C, and lighting was synchronized with natural light. Over the experimental course, water pH was ~7.2, dissolved oxygen > 5.8 mg/L and total ammonia nitrogen was kept < 0.04 mg/L.

### 2.3. Sample Preparation

At the end of the experiment, all fish were fasted for 24 h, then anesthetized with 100 ppm tricane methane sulfonate (MS-222, Sigma, St. Louis, MO, USA) and weighed to determine growth performance. Fifteen fish were randomly selected from each tank and used for blood sampling from the caudal tail vessels; 0.2–0.3 mL was added to heparinized Eppendorf tubes for haematological assays. The remainder of the blood samples were placed at 4 °C for 24 h and then centrifuged at 3500 rpm for 15 min; the serum was removed and stored in liquid nitrogen. The liver and intestine samples were collected from the fish and quick-frozen in liquid nitrogen and then stored at −80 °C for further use.

### 2.4. Haematological and Biochemical Analyses

Blood sample total counts and differential cell types were performed using a TEK 8500 VET automatic blood analyzer (Jiangxi Tekang Technology, Nanchang, China) for white blood cells (WBC), red blood cells (RBC), Hb (HGB), platelets (PLT), mean corpuscular Hb (MCH), mean corpuscular volume (MCV), mean corpuscular Hb concentration (MCHC), monocytes (MON), lymphocytes (LYM) and neutrophils (NEU). Blood sample analyses consisted of 15 replicates in each group. Serum concentrations of albumin (ALB) and activities of alkaline phosphatase (ALP) were measured using a TC6010L autoanalyzer (Tekang Technology. Nanchang, China). Serum analyses consisted of at least six replicates.

### 2.5. Antioxidant Analysis

Frozen tissue specimens (liver and intestine) were pulverized in liquid nitrogen and then suspended in a 9-fold volume (*w/v*) of 0.9% NaCl at 4 °C and then centrifuged at 2500 rpm for 20 min at 4 °C. The supernatants were used for the determination of superoxide dismutase (SOD), catalase (CAT), glutathione reductase (GR), glutathione peroxidase (GPx) and glutathione s-transferase (GST) activities; total antioxidant capacity (T-AOC); and glutathione (GSH) and malondialdehyde (MDA) contents using commercial kits (Jiancheng Bioengineering, Nanjing, China).

Kelch-like ECH-associated protein 1a (Keap1a) amounts were measured using an enzyme-linked immunosorbent assay (ELISA) according to the manufacturer’s instructions (Jiancheng, Nanjing, China). Protein contents were determined using the Coomassie brilliant blue method. All analyses consisted of at least three replicates.

### 2.6. Immunological Analyses

Commercial kits were used for the following enzyme and immunological (ELISA) analyses using methods provided by the manufacturer (Jiancheng, Nanjing, China) These tests included Lysozyme (LZM), acid phosphatase (ACP), ALP and total nitric oxide synthase (T-NOS) activities. ELISA kits were used for complement component 3 (C3), immunoglobulin M (IgM), IL-1β, IL-8, TNF-α, IFN-γ and TGF-β1 measurements. All analyses consisted of at least three replicates.

### 2.7. Gene Expression Measurements

Total RNA was extracted from the intestine and liver using a BioFast Simply P Total RNA Extraction kit (BioFlux, Hangzhou, China), and RNA integrity was examined using 1% agarose gel electrophoresis. RNA was quantified using UV spectroscopy (Nano Drop 2000, Thermo Scientific, Pittsburgh, PA, USA). RNA was reverse transcribed using a PrimeScript RT Kit with gDNA Eraser (Novoprotein, Shanghai, China) following the manufacturer’s instructions. The obtained cDNA templates were then kept frozen at −80 °C until analysis. Primers for quantitative real-time PCR were designed from the largemouth bass genome [32] (Table 2). Real time qPCR was conducted using a NovoStart SYBR qPCR Super Mix Plus (E096-01B, Novoprotein,) using a CFX96 instrument (Bio-Rad, Hercules, CA, USA) following the manufacturer’s protocol. Expression variations were assessed using the 2^−ΔΔCT^ method and β-actin was used as an internal control [17]. All analyses consisted of at least three replicates.

### 2.8. Histomorphometry

Posterior intestine tissue samples were washed with 0.6% saline and fixed in 4% paraformaldehyde for 48 h. After dehydration in a graded ethanol series, the samples were cleared in xylene and embedded in paraffin wax. Histological sections were stained with haematoxylin and eosin (H & E). Micrographs of the intestine were taken at a final magnification of 100× and documented photographically with a digital camera. The images were analysed using K-Viewer (https://kv.kintoneapp.com/en/user/, accessed on 28 August 2022) 1.0 software (1.0.4) for villus height, villus width, muscular thickness and crypt depth measures [33]. All analyses consisted of at least four replicates.

### 2.9. Data Analysis

All results were reported as mean ± SD (standard deviation). One-way analysis of variance (ANOVA) was performed for comparing groups using SPSS 25.0 (IBM, Chicago, IL, USA). Tukey’s multiple interval test was used as a multiple comparison test between different dietary treatments. Moreover, a follow-up trend analysis was conducted using orthogonal polynomial contrasts to determine the significant effects (linear and/or quadratic). *p* < 0.05 indicated a significant difference.

## 3. Results

### 3.1. Growth Performance and Haematological Indices

**Figure 1:**
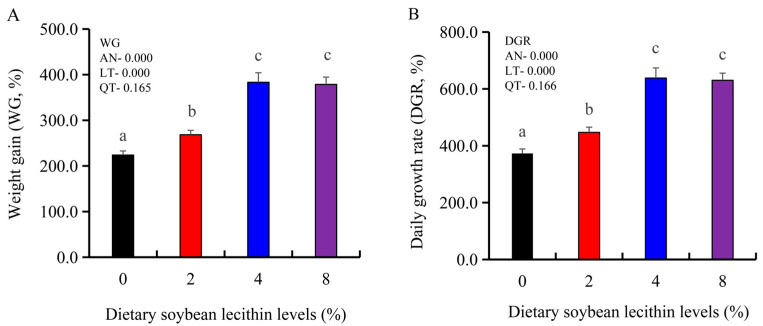
Effect of dietary soybean lecithin on weight gain (%) (**A**) and daily growth rate (%) (**B**) of juvenile largemouth bass. Bars with different letters indicate significant differences (*p* < 0.05), while that with the same letter or no letter superscripts indicate no significant differences (*p* > 0.05), AN: ANOVA, LT: linear trend, QT: quadratic trend.

**Figure 2:**
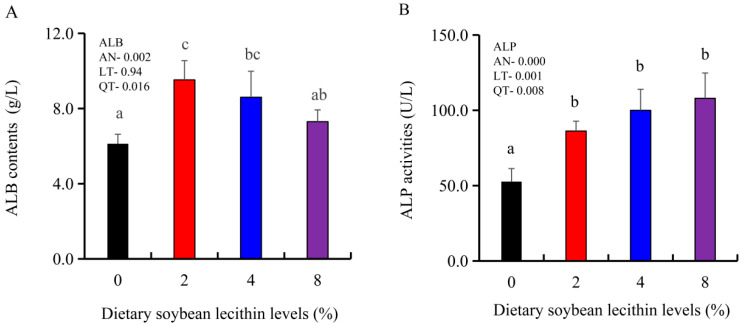
Effect of dietary soybean lecithin on serum albumin (ALB) contents (**A**) and alkaline phosphatase (ALP) activities (**B**) of juvenile largemouth bass. Bars with different letters indicate significant differences (*p* < 0.05), while that with the same letter or no letter superscripts indicate no significant differences (*p* > 0.05), AN: ANOVA, LT: linear trend, QT: quadratic trend.

The growth performance of the juvenile largemouth bass fed our experimental diets for 8 weeks were positively affected by the inclusion of SBL to the feed. The 4 and 8% SBL significantly increased weight gain (WG) and daily growth rate (DGR) of the fish compared with 0 and 2% SBL (*p* < 0.05) (Figure 1). WBC and MON values were also significantly (*p* < 0.05) increased in the 2 and 4% groups compared with the controls, and interestingly, decreased in the 8% group. Similarly, RBC, HGB and PLT levels were the maximum with 4% SBL. MCV and MCH levels displayed an increasing trend positively associated with SBL doses (*p* < 0.05), while MCHC, NEU and LYM values were not altered among these 4 experimental groups (Table 3). However, serum activities of ALP were significantly (*p* < 0.05) greater for the 2 to 8% treatment compared with the controls (0%) that possessed the lowest activities. Serum ALB levels in 2 and 4% SBL were significantly elevated (*p* < 0.05) compared with the controls (Figure 2).

### 3.2. Antioxidant and Oxidant Analyses

#### 3.2.1. Antioxidant and Oxidant Parameters in the Liver

Antioxidative indices in the fish also significantly differed among these test groups. Activities of CAT and GPx were significantly higher in the 4% SBL groups compared with these indices in other three groups (*p* < 0.05). In contrast, MDA contents notably declined with SBL supplementation from 2% to 8% and was the lowest in the 4 and 8% groups (*p* < 0.05). T-AOC, GST activities and GSH levels were significantly higher in liver samples of the 4 and 8% groups (*p* < 0.05). T-SOD and GR activities were enhanced in the 2% group (*p* < 0.05) and then plateaued. However, Keap1a levels were significantly reduced and significantly negatively correlated with SBL doses (*p* < 0.05) (Table 4).

#### 3.2.2. Antioxidant and Oxidant Parameters in the Intestine

In the intestine of our experimental fish, CAT, T-SOD, GPx and GR activities were gradually elevated at 4% SBL (*p* < 0.05) and then declined. GSH contents were the highest in the 8% group compared with the controls (*p* < 0.05). T-AOC activities displayed a significant upward trend to 4 and 8% SBL (*p* < 0.05). GST activities were also significantly increased to the maximum at 4% and then plateaued. In contrast, MDA contents and Keap1a levels were significantly (*p* < 0.05) negatively correlated with SBL doses (Table 5).

### 3.3. Immunological Analysis

#### 3.3.1. Liver Immunological Analysis

Immunological parameters in the liver of our experimental fish were also measured. ACP activities were maximum in the 2 and 4% dietary SBL compared with 0 and 8% diets (*p* < 0.05). Similarly, the fish fed 4% SBL possessed significantly higher levels of C3 and LZM than those in the other three groups (*p* < 0.05). The 4% SBL group also displayed significant decreases for the pro-inflammatory cytokines (IL-8, IL-1β and IFN-γ) (*p* < 0.05), and TNF-α values were also significantly decreased in the 8% SBL groups (*p* < 0.05). Interestingly, the anti-inflammatory cytokine, TGF-β1, displayed a trend that was the opposite as that seen for TNF-α (*p* < 0.05). However, there were no significant differences on T-NOS and ALP activities in the liver among these experimental groups (Table 6 and Table 7).

#### 3.3.2. Intestinal Immunological Analysis

Intestinal immunological and anti-inflammatory indices were significantly up-regulated for LZM, ACP, ALP, T-NOS activities as well as for C3, IgM and TGF-β1. All these indices reached the maximum at 4% dietary SBL (*p* < 0.05). According to these results, the amounts of pro-inflammatory cytokines (IL-8, IL-1β, TNF-α and IFN-γ) were significantly decreased (*p* < 0.05) and negatively correlated with dietary SBL contents (Table 8 and Table 9).

### 3.4. Gene Expression Measurements

#### 3.4.1. Gene Expression in the Liver

**Figure 3:**
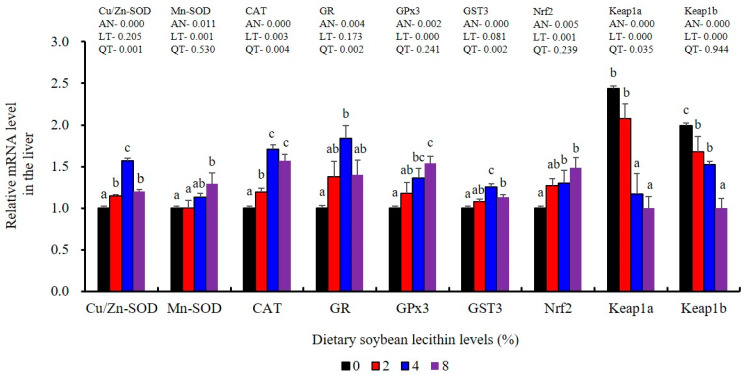
Effect of dietary soybean lecithin on expression levels of antioxidant-related genes in the liver of juvenile largemouth bass. Bars with different letters indicate significant differences (*p* < 0.05), while that with the same letter or no letter superscripts indicate no significant differences (*p* > 0.05), AN: ANOVA, LT: linear trend, QT: quadratic trend.

**Figure 4:**
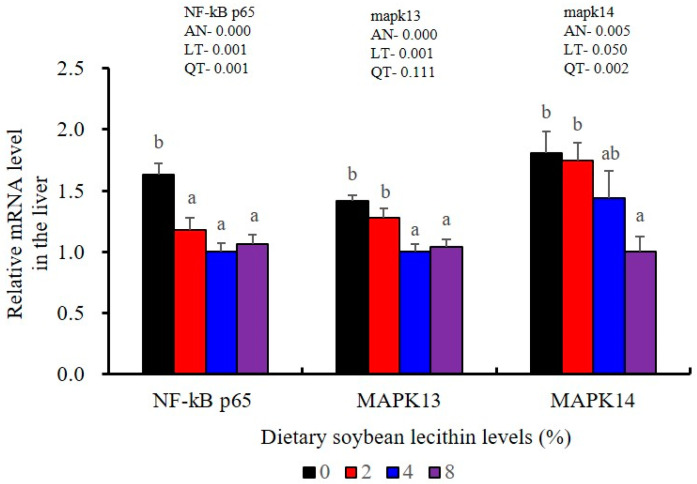
Effects of dietary soybean lecithin on relative gene expression levels of mitogen-activated protein kinase (MAPK) signal pathway in the liver of juvenile largemouth bass. Bars with different letters indicate significant differences (*p* < 0.05), while that with the same letter or no letter superscripts indicate no significant differences (*p* > 0.05), AN: ANOVA, LT: linear trend, QT: quadratic trend.

**Figure 5:**
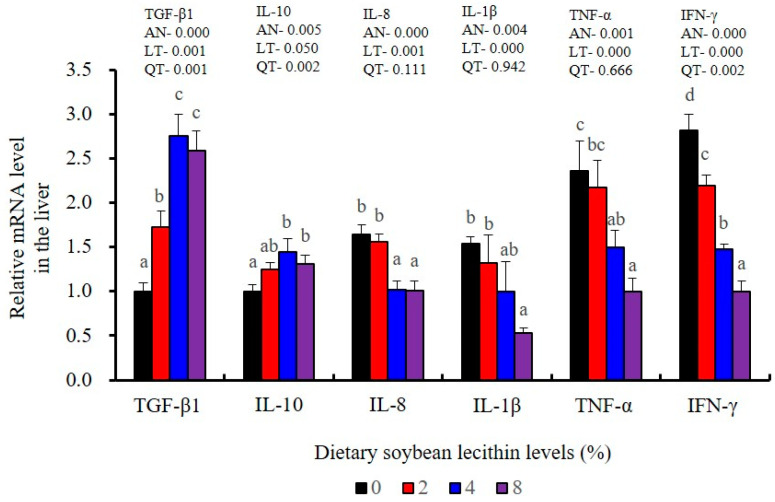
Effect of dietary soybean lecithin on relative gene expression levels of anti-inflammatory and pro-inflammatory cytokines in the liver of juvenile largemouth bass. Bars with different letters indicate significant differences (*p* < 0.05), while that with the same letter or no letter superscripts indicate no significant differences (*p* > 0.05), AN: ANOVA, LT: linear trend, QT: quadratic trend.

**Figure 6:**
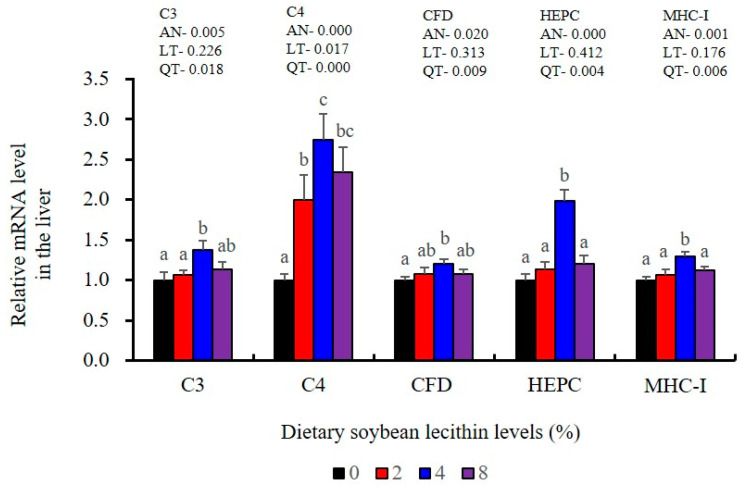
Effect of dietary soybean lecithin on relative gene expression levels of immune-related factors in the liver of juvenile largemouth bass. Bars with different letters indicate significant differences (*p* < 0.05), while that with the same letter or no letter superscripts indicate no significant differences (*p* > 0.05), AN: ANOVA, LT: linear trend, QT: quadratic trend.

The expression of antioxidant-related genes in the liver tissue for Cu/Zn-SOD, CAT, GR and GST3 were significantly enhanced and positively correlated with dietary SBL level and the maximum at 4% SBL (*p* < 0.05) (Figure 3). The mRNA levels of Mn-SOD and GPx3 were increased at 4% dietary SBL and peaked at 8% SBL (*p* < 0.05). Moreover, the 4 to 8% SBL diets significantly up-regulated the expression of Nrf2 compared with controls (*p* < 0.05). In contrast, Keap1a and Keap1b mRNA levels were negatively correlated with dietary SBL doses (*p* < 0.05). As MAPK signalling pathway key genes, NF-κB P65 and MAPK13 were significantly lower at 4% and 8% SBL (*p* < 0.05) (Figure 4). With the SBL dose increasing, MAPK14 mRNA expression levels were only significantly decreased at 8% SBL (*p* < 0.05). The expression amounts of C3, C4, CFD, HEPC, MHC-I, IL-10 and TGF-β1 were all significantly enhanced compared with the controls and reached the maximum at 4% SBL (*p* < 0.05) (Figure 5 and Figure 6). In contrast, the pro-inflammatory cytokines (IL-8, IL-1β, TNF-α and IFN-γ) were significantly lower in the 4% and 8% groups compared with the controls (*p* < 0.05) (Figure 5).

#### 3.4.2. Expression of Immune-Related Genes in the Intestine

**Figure 7:**
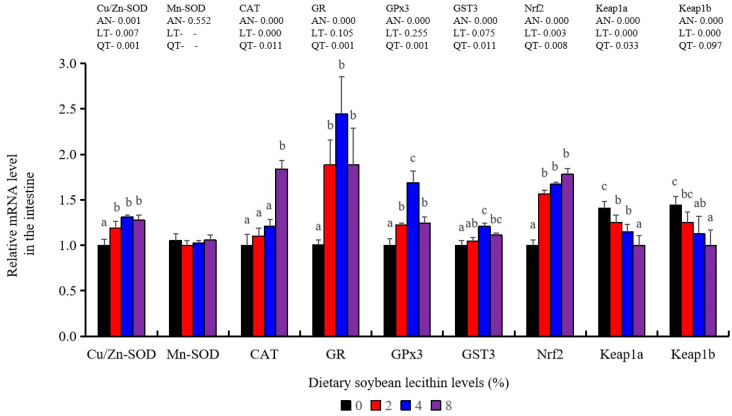
Effect of dietary soybean lecithin on expression levels of antioxidant-related genes in the intestine of juvenile largemouth bass. Bars with different letters indicate significant differences (*p* < 0.05), while that with the same letter or no letter superscripts indicate no significant differences (*p* > 0.05), AN: ANOVA, LT: linear trend, QT: quadratic trend.

Antioxidant-related gene expression assays in the intestine of our experimental fish indicated that GST3 and GPx3 were significantly increased at 4% dietary SBL (*p* < 0.05). CAT mRNA levels were constantly increased and reached the maximum level in the 8% SBL groups (*p* < 0.05). However, Mn-SOD mRNA expression in the intestine was not significant among these four groups. The 2–8% groups displayed significant up-regulation of Cu/Zn-SOD, Nrf2 and GR (*p* < 0.05) versus the control groups but did not differ between the experimental groups. Keap1a and Keap1b levels were significantly decreased and negatively correlated with SBL doses (*p* < 0.05) (Figure 7).

**Figure 8:**
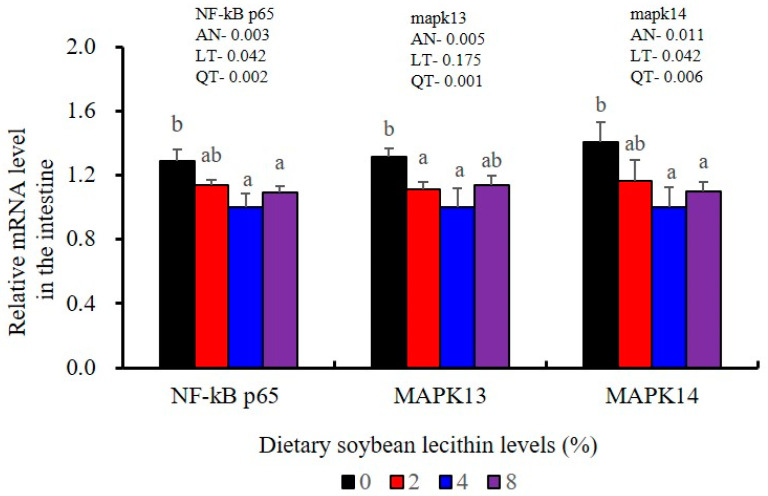
Effects of dietary soybean lecithin levels on relative gene expression levels of mitogen-activated protein kinase (MAPK) signal pathway in the intestine of juvenile largemouth bass. Bars with different letters indicate significant differences (*p* < 0.05), while that with the same letter or no letter superscripts indicate no significant differences (*p* > 0.05), AN: ANOVA, LT: linear trend, QT: quadratic trend.

**Figure 9:**
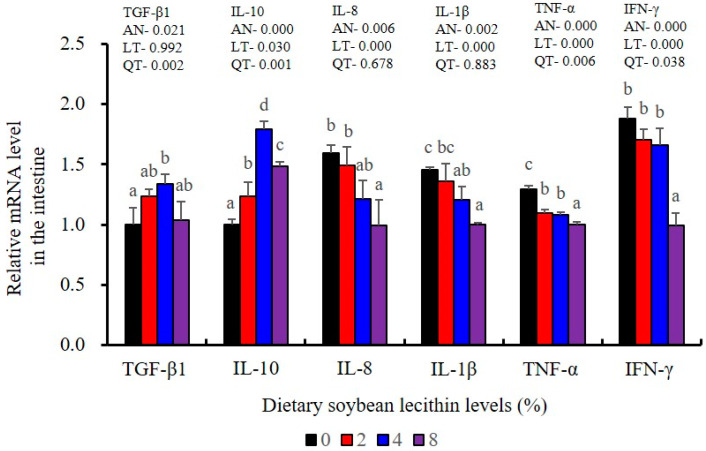
Effect of dietary soybean lecithin on relative gene expression levels of anti-inflammatory and pro-inflammatory cytokines in the intestine of juvenile largemouth bass. Bars with different letters indicate significant differences (*p* < 0.05), while that with the same letter or no letter superscripts indicate no significant differences (*p* > 0.05), AN: ANOVA, LT: linear trend, QT: quadratic trend.

**Figure 10:**
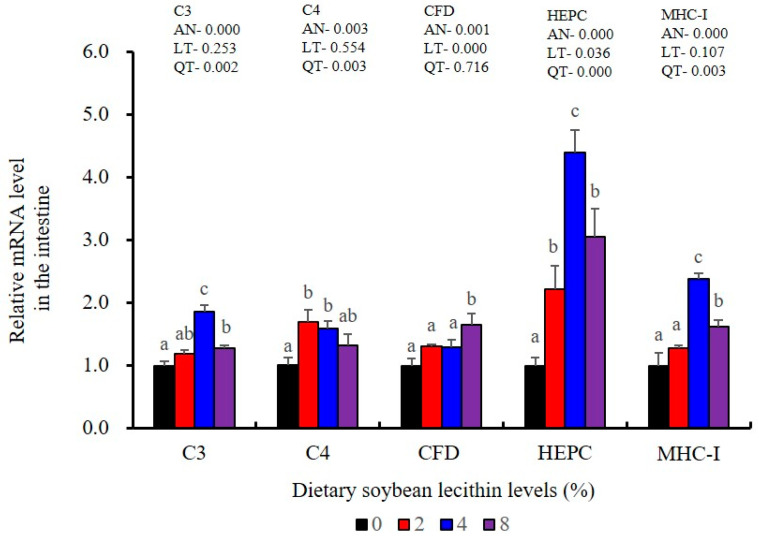
Effect of dietary soybean lecithin on relative gene expression levels of immune-related factors in the intestine of juvenile largemouth bass. Bars with different letters indicate significant differences (*p* < 0.05), while that with the same letter or no letter superscripts indicate no significant differences (*p* > 0.05), AN: ANOVA, LT: linear trend, QT: quadratic trend.

**Figure 11:**
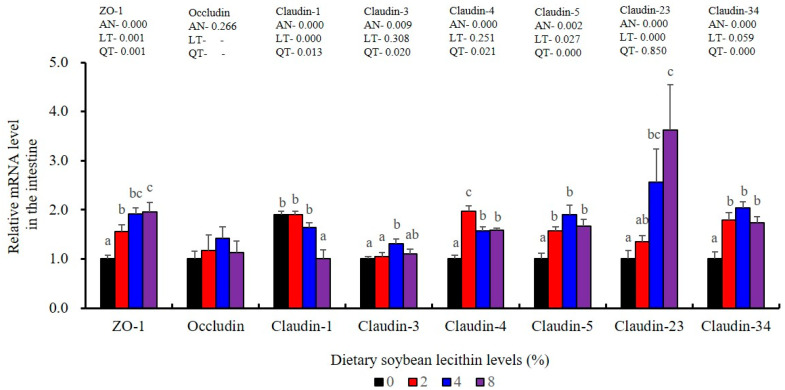
Effects of dietary soybean lecithin on intestinal tight junction-related gene expression levels of juvenile largemouth bass. Bars with different letters indicate significant differences (*p* < 0.05), while that with the same letter or no letter superscripts indicate no significant differences (*p* > 0.05), AN: ANOVA, LT: linear trend, QT: quadratic trend.

**Figure 12:**
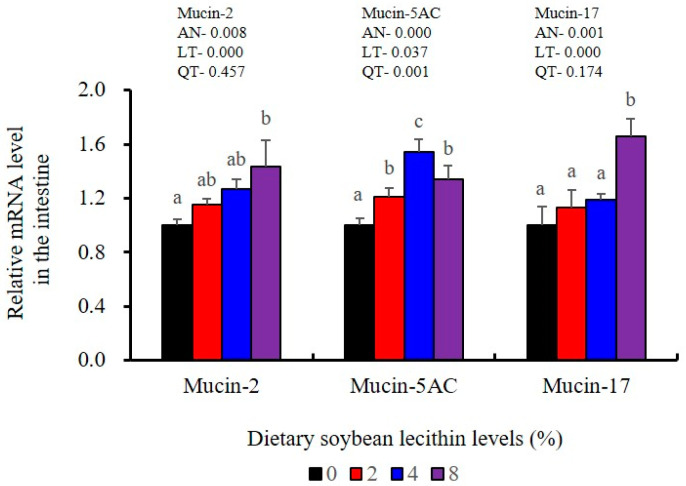
Effects of dietary soybean lecithin levels on intestinal mucins gene expression levels of juvenile largemouth bass. Bars with different letters indicate significant differences (*p* < 0.05), while that with the same letter or no letter superscripts indicate no significant differences (*p* > 0.05), AN: ANOVA, LT: linear trend, QT: quadratic trend.

The expression levels of NF-κB P65, MAPK13 and MAPK14 were all significantly decreased to the lowest at 4% SBL (*p* < 0.05) (Figure 8). In addition, the 4 and 8% SBL groups displayed significantly decreased levels of IL-1β and TNF-α (*p* < 0.05). Although IL-8 and IFN-γ mRNA expression levels decreased with increasing SBL doses, their levels were only significantly lower at 8% SBL (*p* < 0.05) (Figure 9). The mRNA levels for C4 were significantly enhanced compared with controls and especially at the 2 to 4% levels (*p* < 0.05). The 4% SBL group displayed significantly (*p* < 0.05) elevated levels of C3, HEPC, MHC-I, IL-10 and TGF-β1 while CFD level was significantly higher in the 8% group versus the control groups (*p* < 0.05) (Figure 9 and Figure 10). The measurements of mRNA levels for TJs genes in the intestine indicated significant increases for ZO-1, claudin-3, claudin-4, claudin-5, claudin-23 and claudin-34 in the SBL addition groups (*p* < 0.05). In contrast, claudin-1 expression was negatively related with the dose (*p* < 0.05) while the expression of occludin genes was unaffected (Figure 11). Although mRNA levels were constantly improved with increasing SBL doses, mucin-2 and mucin-17 mRNA levels were significantly up-regulated only in the 8% SBL groups compared with the control groups (*p* < 0.05). The mucin-5AC gene in the intestine were significantly up-regulated and reached the highest level in 4% SBL (*p* < 0.05) compared with the control groups (*p* < 0.05) (Figure 12).

### 3.5. Histomorphometry

**Figure 13:**
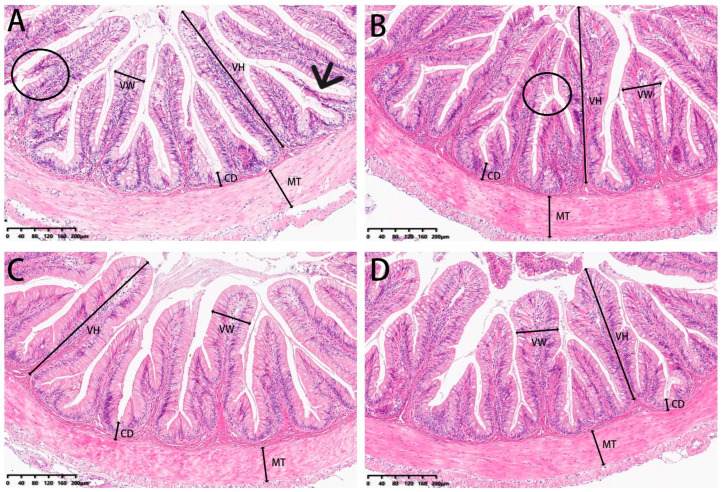
HE staining of the intestine sections of juvenile largemouth bass fed with diets containing 0% (**A**), 2% (**B**), 4% (**C**) and 8% (**D**) SBL (magnification × 100). VH: villus height, VW: villus width, MT: muscular thickness, CD: crypt depth. Circles represent intestinal villi fall off. Black arrow represents villous atrophy.

Our analysis of experimental fish also included examinations of the microstructure of intestinal sections (Figure 13). Villi height and muscle thickness were all decreased in the 0 and 2% SBL groups compared with the 4 and 8% groups (*p* < 0.05). Crypt depths and villus widths were not significant (Table 10). The morphology was characterized by detached villi and villous atrophy in the 0 and 2% SBL groups. In contrast, the morphology of the intestine in the 4 and 8% groups was well-developed with increased villi heights and muscular thickness that was the highest in the 4% SBL group (Figure 13).

## 4. Discussion

As a functional lipid source, SBL has been widely used in the compound feeds of terrestrial and aquatic animals. Many studies have found that dietary SBL could influence the growth performances in different animals. For example, adequate dietary SBL could increase the growth in Caspian brown trout (*S. trutta caspius*) [9], common carp (*C. carpio*) [10], stellate sturgeon (*A. stellatus*) [11], rock bream (*O. fasciatus*) [12], sea urchin (*Strongylocentrotus intermedius*) [34], pacific white shrimp (*Litopenaeus vannamei*) [35], cattle [1] and chickens [3]. Consistent with these studies, it was found that SBL added at 4 and 8% significantly increased WG and DGR for the largemouth bass, which indicated that SBL dietary supplementation have had positive effects on the growth of this species. However, other studies have found no significant growth effects of SBL addition in golden mahseer (*T. putitora*) [6] and channel catfish (*I. punctatus*) [13]. These differences may be related to the different fish species, developmental periods and rearing conditions [6,8,9,10,11,12,13].

Haematological parameters are important indicators of the health status and nutritional conditions of aquatic animals. Consistent with previous results in trout [9] and sturgeon [11], it was found that 4% SBL elevated WBC and MON levels in blood, indicating enhanced immunity [36]. It was also found that 4% SBL increased RBC, HGB, PLT, MCV and MCH levels suggesting that SBL could reduce inflammation and enhance the oxygen-carrying capacity in largemouth bass, which was consistent with the results in Nile tilapia (*O. niloticus*) [15,36]. ALB content is also an effective indicator of liver and immune dysfunction and was also significantly increased with dietary SBL supplementation, which is similar to the results in rainbow trout (*Oncorhynchus mykiss*) [37]. ALP activities were significantly boosted in the serum and intestine, similar to the results of previous studies in largemouth bass (*M. salmoides*) [38]. Therefore, these results suggest that the addition of adequate SBL could enhance the immune status by improving the related haematology and serum biochemistry indices in juvenile largemouth bass.

It is well-known that ROS could be constantly produced during the metabolism of all nutrients and influence the activities of antioxidant enzymes in aerobic organisms. In the current study, SBL addition enhanced antioxidant capabilities by increasing T-SOD, T-AOC, CAT, GPx and GSH. These results were similar to the results reported in golden mahseer (*T. putitora*) [6], Caspian brown trout (*S. trutta caspius*) [9], common carp (*C. carpio*) [10] and sea urchin (*S. intermedius*) [34]. These data suggest that adequate SBL alleviates ROS toxicity via increasing antioxidant capabilities [39]. In both the liver and the intestine, GST activities were positively correlated with SBL dose while MDA content presented the opposite trend as GST. These data were consistent with SBL reducing the damage to the body caused by oxidative stress. Interestingly, Nrf2 and its downstream targets Cu/Zn-SOD, CAT, GR, GST and GPx3 were all up-regulated and positively associated with SBL doses, while Keap1a and Keap1b expression displayed the opposite effect. These results were similar to the results of previous studies in grass carp (*C. idella*) [40] and *Sillago sihama* [19]. Nrf2/Keap1 signalling is a primary regulator of the antioxidant response where Nrf2 binds to Keap1 to maintain an inhibitory state under normal physiological conditions [41]. Under oxidative stress, this association is broken and Nrf2 is translocated to the nucleus to activate antioxidant enzyme genes [21]. Therefore, dietary SBL could promote the expression of relevant antioxidant genes via Nrf2/Keap1 signalling that serve to minimize oxidative damage in juvenile largemouth bass.

LZM and ACP are additional diagnostic indexes used to evaluate the health status in fish [25]. Complement C3 release enhances phagocytosis as well as the macrophage respiratory burst that also includes NO production that serves a key immune function in fish [42]. The elevated LZM, ACP and complement components in fish fed SBL have been previously reported in stellate sturgeon (*A. stellatus*) [11], Caspian brown trout (*S. trutta caspius*) [9] and common carp (*C. carpio*) [10]. T-NOS activities were also significantly increased in the intestine for the 4% SBL group, and similar findings were reported in largemouth bass (*M. salmoides*) fed adequate fibre [43]. Similarly, IgM levels were also significantly enhanced in the intestine of our experimental fish fed 4% SBL and were consistent with the results for Caspian brown trout (*S*. *trutta caspius*) [9]. IgM is a primary component of fish humoral response and is essential for immune clearance of pathogens [44]. HEPC also participates in this process as an antimicrobial peptide and enhances phagocytic endocytosis of pathogens [45], and MHC-I plays a pivotal role in antigen presentation [46]. The elevated expression levels of HEPC, MHC-I, C3, C4 and CFD in our experimental fish fed adequate dietary SBL were consistent with results in black carp (*Mylopharyngodon piceus*) [47]. Together, these results indicated that adequate dietary SBL improved systemic and mucosal immunity via enhancing direct defence effectors in the intestine of juvenile largemouth bass.

Furthermore, chronic inflammation is associated with impaired health status [25]. Inflammatory responses are primarily regulated by pro-inflammatory cytokines (IL-1β, TNF-α, IFN-γ and IL-8) and anti-inflammatory cytokines (IL-10 and TGF-β1) [22]. We found that adequate dietary SBL inhibited the expression levels of these four inflammatory cytokines (IL-1β, TNF-α, IFN-γ and IL-8) and promoted expression of TGF-β1, which is in agreement with previous results in largemouth bass (*M. salmoides*) [27] and rainbow trout (*O*. *mykiss*) [37]. These cytokines levels were also mirrored at the gene expression levels, indicating that SBL could modulate the critical p38 MAPK/NF-κB inflammatory signalling pathway [23]. In our study, compared with the controls, 4 and 8% dietary SBL significantly down-regulated the expression of MAPK13, MAPK14 and NF-κB P65 in the liver and intestine of largemouth bass, which is consistent with previous studies in this fish [5] and grass carp (*C. idella*) [40]. The overall effect of adequate SBL was to enhance innate immunity while inhibiting a hyperactive p38 MAPK/ NF-κB responses.

The intestinal tract is the primary barrier that limits the entry of anti-nutritional factors and pathogenic substances. Largemouth bass fed with the trace mineral supplement azomite displayed enhanced barrier health [48], which is similar with our results in 4 and 8% SBL. Well-developed intestinal structures with increased villus height and muscular thickness in our results indicated better intestinal health for largemouth bass [33]. However, there were no effects associated with soybean oil replacement by SBL in the intestinal tract of broiler chickens [3], which might be due to differences between terrestrial and aquatic animals. We also found that the integrity and selective permeability of the intestinal cell barrier is maintained in epithelial cells linked via transmembrane TJs including occludin, zonula occludens-1 (ZO-1) and claudin [29]. Similar with previous results in largemouth bass (*M. salmoides*) fed phospholipid and starch [5,27], our present study found that 4 and 8% dietary SBL elevated expression of ZO-1, claudin-3, claudin-4, claudin-5, claudin-23 and claudin-34, while suppressed expression of claudin-1, which indicated that higher levels of TJs could improve intestinal barrier functions and integrity [27,28,29,30]. Meanwhile, the expression of mucin-2, mucin-5AC and mucin-17 was also enhanced with the higher doses of SBL, suggesting SBL could enhance the mucus barrier [26]. Taken together, these results indicate that adequate SBL supplement resulted in a positive and protective effect in maintaining intestinal barrier function and increased mucin contents [43].

In conclusion, 4% dietary SBL could improve growth as well as haematological and serum biochemical indicators of health. Adequate dietary SBL could up-regulate antioxidant capabilities by increasing antioxidant enzyme activities through the Nrf2/Keap1 pathway, boost immunity by raising direct defensive effectors and alleviate the inflammatory responses through p38 MAPK/NF-κB regulation. By modulating the expression of TJs and mucin-5AC, adequate SBL also improved intestinal barrier properties in largemouth bass. These findings supplied the benefits of adequate SBL used in functional artificial feed of largemouth bass.

## Figures and Tables

**Table 1:** Ingredient and proximate composition of the experimental diets (on dry weight basis).

Ingredients (%)	Diets (%)			
	0	2	4	8
Defatted fish meal ^a^	20.00	20.00	20.00	20.00
Casein ^b^	35.00	35.00	35.00	35.00
Gelatine ^c^	5.00	5.00	5.00	5.00
Soybean oil ^d^	10.00	8.00	6.00	2.00
Soybean lecithin ^e^	0.00	2.00	4.00	8.00
Dextrin ^c^	10.00	10.00	10.00	10.00
Mineral premix ^f^	2.40	2.40	2.40	2.40
Vitamin premix ^g^	1.20	1.20	1.20	1.20
Choline chloride ^h^	0.40	0.40	0.40	0.40
Microcrystalline cellulose ^c^	16.00	16.00	16.00	16.00
Proximate analysis (%)				
Moisture	6.49	6.64	6.61	6.76
Crude protein	50.67	50.59	50.65	50.53
Crude lipid	9.58	9.53	9.57	9.49
Crude ash	5.61	5.57	5.67	5.78
Gross energy (MJ kg^−1^)	17.43	17.34	17.38	17.41

^a^ Defatted fish meal, Pesquera Diamante S.A. crude protein 76.7%; crude lipid 1.8%. ^b^ Casein obtained from Gansu Hualing Dairy Co., Ltd., Lanzhou, China; crude protein 80.56%. ^c^ Gelatine, Dextrin and Microcrystalline cellulose, obtained from Sinopharm Chemical Reagent Co., Ltd., Shanghai, China, crude protein 99.76%. ^d^ Soybean oil, produced using oil press. ^e^ Soybean lecithin, Jiangsu Yuanshengyuan Biological Engineering Co., Ltd., Nanjing, China. ^f^ Mineral Premix (mg kg^−1^ diet): Na_2_SeO_3_, 1.2 mg; KI, 2 mg; CoCl_2_·6H_2_O, 13 mg; CuSO_4_·5H_2_O, 5 mg; FeSO_4_·7H_2_O, 70 mg; ZnSO_4_·7H_2_O, 100 mg; MnSO_4_·H_2_O, 45 mg; MgSO_4_·7H_2_O, 130 mg; NaCl, 50 mg. ^g^ Vitamin Premix (mg kg^−1^ diet): vitamin A, 35 mg; vitamin D, 6 mg; vitamin C, 1000 mg; vitamin E, 300 mg; thiamine, 30 mg; riboflavin, 50 mg; pyridoxine HCl, 20 mg; vitamin B12, 0.1 mg; vitamin K3, 10 mg; inositol, 800 mg; pantothenic acid, 60 mg; folic acid, 20 mg; niacin acid, 200 mg; biotin, 60 mg. ^h^ Zhejiang Yixing Feed Group Co. Ltd., Jiaxing, China.

**Table 2:** Primer sequences for real-time PCR analysis.

Gene	Primers	Primer Sequence (5′–3′)	Reference
Cu/Zn-SOD	F	AGGTAGTGAGGCTTGTCTGC	XM_038708943.1
	R	TCGCCCTCCTGCTCAAAATA	
Mn-SOD	F	AAACCTGGTAGCTGCATCCA	XM_038727054.1
	R	TGTGGTTAATGTGGCCTCCT	
CAT	F	CTGGCGAATGTGTCTACTGC	XM_038704976.1
	R	CTTGGTCAGATCGAAGGGGT	
GPx3	F	GCCTTTCCCACCAACCAATT	XM_038699914.1
	R	TCGTTGATCTTCAGAGGCCC	
GR	F	TGTAAAACGGACAGAGGGCT	XM_038700350.1
	R	CTCGCCGATGTTCAATCCAG	
GST3	F	CGTGGAGAGATGGACGTTCT	XM_038729946.1
	R	CTTGTCGCTGTACATGGTGG	
Keap1a	F	TTCAGACGGCAGGAGATGTT	XM_038728593.1
	R	ATGTGGGAGGTGTAGGCAAA	
Keap1b	F	AGCCGGGTACAATGCTTAGT	XM_038713665.1
	R	GGAGTTTGGTTCGTTGGCTT	
Nrf2	F	AGCGAACTGGACTCACTGAA	XM_038720536.1
	R	CTGCTGGAGGGAGTAGTCTG	
C3	F	ATGACCAACTGCAACGTGAC	XM_038714039.1
	R	CTCGCTGGCTTTCAATCTCC	
C4	F	CTCTAAGACAGGCACGTGGA	XM_038711699.1
	R	CAAACTGGCAGTGGAAAGCT	
CFD	F	GGTGCCCATTCTCTGAGTGA	XM_038729044.1
	R	CCTCTGCATTTGTGTCAGGG	
HEPC	F	TTGTGGTGCTCTTTTGGTGG	XM_038710826.1
	R	CCGCAACTGGAGTGTCATTG	
MHC-I	F	TCATTGCTGGGGTGTTTGTG	XM_038725863.1
	R	ACTACATCTGGACCTTGGGC	
TGF-β1	F	GCTCAAAGAGAGCGAGGATG	XM_038693206.1
	R	TCCTCTACCATTCGCAATCC	
IL-10	F	CGGCACAGAAATCCCAGAGC	XM_038696252.1
	R	CAGCAGGCTCACAAAATAAACATCT	
IL-1β	F	TTCAAACTGCAGGCCATCAC	XM_038733429.1
	R	CAGCTTTACCGCCTTCCATG	
IL-8	F	GCTGCAGAGGTTGAATGACTAC	XM_038713529.1
	R	TCCAATGGGCCTTTTCTCCT	
TNF-α	F	TGTGACGGAGACCAGATGAC	XM_038699437.1
	R	CAGACCGGCTTCAACAAACA	
IFN-γ	F	AGATCAGAGGCTTTCAAATCCC	XM_026298863.1
	R	CAACATGTGGCTAATCAGCTT	
NF-κB P65	F	GCGAGCCCCTTCATAGAGAT	XM_038730802.1
	R	ACCAATGAGATGCGAACACG	
MAPK13	F	CTGCTTGAGAAGATGCTGGTT	XM_038723459.1
	R	AGGCTGTCGAAATATGGGTG	
MAPK14	F	TTCGATGGAGACGAGATGG	XM_038696748.1
	R	GAGATGAATGACCGCAGGC	
ZO-1	F	ATCTCAGCAGGGATTCGACG	XM_038701018.1
	R	CTTTTGCGGTGGCGTTGG	
Occludin	F	ATGTGCCAGAACCTGTACCA	XM_038715419.1
	R	GTTTCCCGTAACGCCAGATC	
Claudin-1	F	CCAGGGAAGGGGAGCAATG	XM_038713307.1
	R	GCTCTTTGAACCAGTGCGAC	
Claudin-3	F	GGCTTTGCTCATCTCCATCG	XM_038693400.1
	R	GATTATGGTGTTGGCCGACC	
Claudin-4	F	TAATCGCTATGGTGGGAGCC	XM_038708626.1
	R	GCCCCGATCTCCATCTTCTG	
Claudin-5	F	TGGGACTATCGATGTCGATA	XM_038704228.1
	R	CCACAAGCCGTCCCAGATAG	
Claudin-23	F	GTCCTGACCTCATTGCTCCT	XM_038729173.1
	R	TACACCCCAAGAGCAGTCAG	
Claudin-34	F	TGCATTTCCTGTGTTACCGC	XM_038715311.1
	R	TAGGATTGTCTCTGCCGTGG	
Mucin-2	F	TTGTAATGAGGCCGGTGTCT	XM_038706114.1
	R	CCAGGTGGGGTATAGGGTTG	
Mucin-5AC	F	TGGGCCTTCTAGAGTCTCCT	XM_038696526.1
	R	CTGATTGGTTTAGCGCCCTC	
Mucin-17	F	AAGTCCACATCCAGTCCCAG	XM_038725272.1
	R	TCCTTGTCCTGTCCTTGTCC	
β-actin	F	TTCACCACCACAGCCGAAAG	XM_038695351.1
	R	TCTGGGCAACGGAACCTCT	

F: forward, R: reverse. Cu/Zn-Superoxide dismutase, Cu/Zn-SOD; Mn-Superoxide dismutase, Mn-SOD; catalase, CAT; glutathione peroxidase 3, GPx3; glutathione reductase, GR; glutathione S-transferase 3, GST3; kelch-like ECH-associated protein 1a, Keap1a; kelch-like ECH-associated protein 1b, Keap1b; NF-E2-related factor 2, Nrf2; complement component 3, C3; complement component 4, C4; complement factor D, CFD; hepcidin, HEPC; major histocompatibility complex class I, MHC-I; interleukin 10, IL-10; transforming growth factor beta 1, TGF-β1; interleukin -1β, IL-1β; interleukin 8, IL-8; tumour necrosis factor-α, TNF-α; interferon-γ, IFN-γ; nuclear factor-kappa B p65, NF-κB P65; mitogen-activated protein kinase 13, MAPK13; mitogen-activated protein kinase 14, MAPK14; zonula occludens-1, ZO-1.

**Table 3:** Haematological parameters of juvenile largemouth bass fed diet trials.

	Lecithin (%) in Diets						
Parameters	0	2	4	8	AN	LT	QT
WBC (10^9^/L)	171.30 ± 7.79 ^a^	187.70 ± 6.26 ^b^	190.80 ± 3.87 ^b^	175.21 ± 3.99 ^a^	0.001	0.929	0.000
RBC (10^12^/L)	2.73 ± 0.09 ^ab^	2.84 ± 0.17 ^ab^	3.00 ± 0.14 ^b^	2.66 ± 0.16 ^a^	0.027	0.533	0.006
HGB (g/L)	86.75 ± 1.94 ^a^	89.00 ± 3.54 ^ab^	93.00 ± 0.91 ^b^	90.88 ± 3.20 ^ab^	0.031	0.061	0.036
PLT (10^9^/L)	123.50 ± 9.57 ^ab^	132.38 ± 11.88 ^ab^	140.88 ± 7.38 ^b^	116.00 ± 7.35 ^a^	0.013	0.324	0.002
MCV (fL)	125.68 ± 3.05 ^a^	134.65 ± 6.73 ^ab^	146.73 ± 7.26 ^b^	161.73 ± 5.21 ^c^	0.000	0.000	0.505
MCH (pg)	30.40 ± 0.86 ^a^	32.03 ± 0.93 ^ab^	32.58 ± 0.53 ^b^	33.53 ± 0.89 ^b^	0.001	0.000	0.120
MCHC (g/L)	224.50 ± 8.34	221.50 ± 7.77	220.00 ± 11.07	217.50 ± 7.59	0.727	-	-
NEU (10^9^/L)	28.95 ± 2.75	27.73 ± 1.67	27.59 ± 1.49	24.89 ± 1.95	0.081	-	-
LYM (10^9^/L)	134.90 ± 3.36	137.21 ± 4.00	140.32 ± 1.41	136.97 ± 2.49	0.052	-	-
MON (10^9^/L)	27.72 ± 2.50 ^ab^	28.84 ± 1.61 ^b^	29.64 ± 1.40 ^b^	25.21 ± 1.08 ^a^	0.018	0.078	0.008

Note: ^a–c^ Values in the same row with different letters indicate significant differences (*p* < 0.05), while that with the same letter or no letter superscripts indicate no significant differences (*p* > 0.05). AN: ANOVA, LT: linear trend, QT: quadratic trend.

**Table 4:** Effects of dietary soybean lecithin on the antioxidative and oxidative indices in the liver of largemouth bass.

	Lecithin (%) in Diets						
Parameters	0	2	4	8	AN	LT	QT
T-SOD (U/mgprot)	507.67 ± 10.41 ^a^	575.54 ± 15.07 ^b^	630.14 ± 34.80 ^b^	603.26 ± 28.86 ^b^	0.002	0.019	0.001
CAT (U/mgprot)	15.37 ± 0.28 ^a^	23.97 ± 0.72 ^c^	26.47 ± 0.98 ^d^	20.77 ± 0.75 ^b^	0.000	0.277	0.000
GPX (U/mgprot)	51.96 ± 1.52 ^a^	63.75 ± 3.32 ^b^	88.23 ± 3.58 ^d^	80.22 ± 0.70 ^c^	0.000	0.004	0.001
GR (U/gprot)	7.55 ± 0.33 ^a^	9.18 ± 0.50 ^b^	9.46 ± 0.79 ^b^	9.38 ± 0.70 ^b^	0.015	0.033	0.017
GST (U/mgprot)	39.69 ± 1.29 ^a^	41.97 ± 1.52 ^a^	48.69 ± 1.34 ^b^	49.08 ± 1.07 ^b^	0.000	0.000	0.034
GSH (μmol/gprot)	58.09 ± 1.13 ^a^	59.16 ± 3.07 ^a^	76.88 ± 1.55 ^b^	111.12 ± 8.32 ^b^	0.000	0.000	0.015
Keap1a (ng/mgprot)	151.58 ± 3.52 ^b^	147.46 ± 7.02 ^ab^	137.52 ± 6.53 ^a^	136.44 ± 2.85 ^a^	0.000	0.002	0.061
T-AOC (μmol/gprot)	30.83 ± 1.78 ^a^	30.66 ± 2.16 ^a^	90.99 ± 7.00 ^b^	117.31 ± 4.70 ^c^	0.000	0.000	0.679
MDA (nmol/mgprot)	1.46 ± 0.12 ^c^	0.92 ± 0.10 ^b^	0.52 ± 0.04 ^a^	0.68 ± 0.03 ^a^	0.000	0.006	0.000

Note: ^a–c^ Values in the same row with different letters indicate significant differences (*p* < 0.05), while that with the same letter or no letter superscripts indicate no significant differences (*p* > 0.05). AN: ANOVA, LT: linear trend, QT: quadratic trend.

**Table 5:** Effects of dietary soybean lecithin on the antioxidative and oxidative indices in the intestine of largemouth bass.

	Lecithin (%) in Diets						
Parameters	0	2	4	8	AN	LT	QT
T-SOD (U/mgprot)	128.31 ± 7.01 ^a^	146.30 ± 2.59 ^b^	196.84 ± 11.31 ^d^	165.17 ± 2.06 ^c^	0.000	0.056	0.002
CAT (U/mgprot)	14.31 ± 1.05 ^a^	20.82 ± 1.94 ^b^	27.71 ± 1.02 ^c^	22.39 ± 0.65 ^b^	0.000	0.050	0.000
GPx (U/mgprot)	67.89 ± 3.07 ^a^	95.10 ± 3.49 ^b^	114.24 ± 4.61 ^c^	97.81 ± 0.74 ^b^	0.000	0.219	0.000
GR (U/gprot)	59.14 ± 2.46 ^a^	84.51 ± 5.32 ^b^	105.38 ± 8.67 ^c^	56.80 ± 3.99 ^a^	0.000	0.745	0.000
GST (U/mgprot)	191.24 ± 14.27 ^a^	261.73 ± 12.84 ^b^	352.95 ± 10.54 ^c^	347.04 ± 32.31 ^c^	0.000	0.001	0.001
GSH (μmol/gprot)	49.86 ± 4.02 ^a^	72.25 ± 4.75 ^b^	116.63 ± 8.96 ^c^	182.59 ± 9.71 ^d^	0.000	0.000	0.526
Keap1a (ng/mgprot)	167.90 ± 9.84 ^b^	146.01 ± 7.93 ^a^	141.10 ± 6.07 ^a^	147.43 ± 5.10 ^a^	0.000	0.087	0.004
T-AOC (μmol/gprot)	18.00 ± 1.393 ^a^	18.67 ± 2.052 ^a^	47.05 ± 2.506 ^b^	67.69 ± 3.015 ^c^	0.000	0.000	0.794
MDA (nmol/mgprot)	8.79 ± 0.30 ^c^	7.83 ± 0.10 ^b^	5.25 ± 0.40 ^a^	5.47 ± 0.42 ^a^	0.000	0.001	0.008

Note: ^a–c^ Values in the same row with different letters indicate significant differences (*p* < 0.05), while that with the same letter or no letter superscripts indicate no significant differences (*p* > 0.05). AN: ANOVA, LT: linear trend, QT: quadratic trend.

**Table 6:** Effects of dietary soybean lecithin on the immune parameters in the liver of largemouth bass.

	Lecithin (%) in Diets						
Parameters	0	2	4	8	AN	LT	QT
LZM (U/mgprot)	29.49 ± 2.08 ^a^	33.27 ± 2.28 ^ab^	40.32 ± 1.89 ^c^	37.61 ± 2.92 ^bc^	0.002	0.016	0.010
ACP (U/gprot)	143.61 ± 13.68 ^a^	249.80 ± 14.57 ^c^	254.52 ± 16.34 ^c^	190.50 ± 7.86 ^b^	0.000	0.584	0.000
ALP (U/gprot)	49.61 ± 1.11	49.10 ± 3.84	49.42 ± 2.30	48.42 ± 1.79	0.937	-	-
T-NOS (U/mgprot)	3.60 ± 0.31	3.84 ± 0.22	3.82 ± 0.24	3.91 ± 0.36	0.600	-	-
C3 (μg/mgprot)	87.89 ± 1.58 ^a^	91.69 ± 3.07 ^a^	113.96 ± 3.16 ^c^	102.62 ± 4.57 ^b^	0.000	0.043	0.014

Note: ^a–c^ Values in the same row with different letters indicate significant differences (*p* < 0.05), while that with the same letter or no letter superscripts indicate no significant differences (*p* > 0.05). AN: ANOVA, LT: linear trend, QT: quadratic trend.

**Table 7:** Effects of dietary soybean lecithin on the anti-inflammatory and pro-inflammatory cytokines in the liver of largemouth bass.

	Lecithin (%) in Diets						
Parameters	0	2	4	8	AN	LT	QT
TGF-β1 (ng/mgprot)	0.20 ± 0.01 ^a^	0.20 ± 0.01 ^a^	0.27 ± 0.03 ^b^	0.31 ± 0.02 ^b^	0.002	0.004	0.007
IL-8 (ng/gprot)	42.38 ± 1.33 ^b^	38.73 ± 2.71 ^ab^	34.31 ± 3.19 ^a^	36.22 ± 1.92 ^ab^	0.016	0.033	0.019
IL-1β (ng/gprot)	15.56 ± 0.98 ^b^	14.70 ± 0.82 ^ab^	12.31 ± 0.81 ^a^	13.28 ± 1.10 ^ab^	0.012	0.036	0.040
TNF-α (ng/gprot)	38.73 ± 0.94 ^c^	37.37 ± 0.97 ^bc^	35.06 ± 1.11 ^ab^	34.29 ± 1.32 ^a^	0.004	0.001	0.144
IFN-γ (ng/gprot)	37.69 ± 0.59 ^c^	36.37 ± 0.46 ^c^	29.70 ± 1.52 ^a^	33.10 ± 0.19 ^b^	0.000	0.038	0.010

Note: ^a–c^ Values in the same row with different letters indicate significant differences (*p* < 0.05), while that with the same letter or no letter superscripts indicate no significant differences (*p* > 0.05). AN: ANOVA, LT: linear trend, QT: quadratic trend.

**Table 8:** Effects of dietary soybean lecithin on the immune parameters in the intestine of largemouth bass.

	Lecithin (%) in Diets						
Parameters	0	2	4	8	AN	LT	QT
LZM (U/mgprot)	203.42 ± 12.77 ^a^	217.28 ± 7.97 ^ab^	278.79 ± 13.72 ^c^	241.49 ± 15.16 ^b^	0.000	0.081	0.011
ACP (U/gprot)	141.01 ± 2.37 ^a^	177.22 ± 8.89 ^b^	303.27 ± 11.91 ^c^	150.17 ± 1.85 ^a^	0.000	0.786	0.001
ALP (U/gprot)	296.56 ± 5.42 ^a^	322.56 ± 8.05 ^b^	693.55 ± 6.86 ^d^	434.08 ± 5.80 ^c^	0.000	0.176	0.010
T-NOS (U/mgprot)	5.53 ± 0.35 ^a^	7.24 ± 0.45 ^b^	9.10 ± 0.77 ^c^	7.94 ± 0.51 ^bc^	0.000	0.031	0.000
C3 (μg/mgprot)	93.96 ± 3.33 ^a^	105.79 ± 4.97 ^b^	137.75 ± 3.56 ^c^	105.41 ± 5.61 ^ab^	0.000	0.376	0.001
IgM (μg/mgprot)	167.73 ± 7.63 ^a^	178.06 ± 6.54 ^a^	241.60 ± 10.34 ^b^	176.95 ± 11.76 ^a^	0.000	0.568	0.004

Note: ^a–c^ Values in the same row with different letters indicate significant differences (*p* < 0.05), while that with the same letter or no letter superscripts indicate no significant differences (*p* > 0.05). AN: ANOVA, LT: linear trend, QT: quadratic trend.

**Table 9:** Effects of dietary soybean lecithin on the anti-inflammatory and pro-inflammatory cytokines in the intestine of largemouth bass.

	Lecithin (%) in Diets						
Parameters	0	2	4	8	AN	LT	QT
TGF-β1 (ng/mgprot)	1.85 ± 0.05 ^a^	2.08 ± 0.18 ^ab^	2.35 ± 0.19 ^b^	2.03 ± 0.11 ^a^	0.019	0.337	0.004
IL-8 (ng/gprot)	68.10 ± 1.50 ^c^	63.42 ± 4.91 ^bc^	50.57 ± 1.93 ^a^	59.75 ± 2.85 ^b^	0.001	0.114	0.005
IL-1β (ng/gprot)	17.34 ± 0.68 ^c^	16.29 ± 0.99 ^bc^	14.27 ± 0.65 ^ab^	12.36 ± 1.34 ^a^	0.001	0.000	0.584
TNF-α (ng/gprot)	37.64 ± 1.01 ^b^	35.01 ± 2.06 ^b^	28.50 ± 1.80 ^a^	26.29 ± 0.95 ^a^	0.000	0.000	0.079
IFN-γ (ng/gprot)	28.59 ± 0.82 ^b^	23.84 ± 1.62 ^a^	22.68 ± 1.32 ^a^	22.44 ± 1.05 ^a^	0.001	0.006	0.003

Note: ^a–c^ Values in the same row with different letters indicate significant differences (*p* < 0.05), while that with the same letter or no letter superscripts indicate no significant differences (*p* > 0.05). AN: ANOVA, LT: linear trend, QT: quadratic trend.

**Table 10:** The effects of soybean lecithin on the intestinal morphology of juvenile largemouth bass.

	Lecithin (%) in Diets						
Parameters	0	2	4	8	AN	LT	QT
Villi height (μm)	456.96 ± 17.21 ^a^	459.69 ± 22.44 ^a^	508.00 ± 15.11 ^b^	463.15 ± 9.68 ^a^	0.043	0.704	0.088
Villi width (μm)	128.45 ± 15.54	131.00 ± 13.11	145.00 ± 10.52	133.11 ± 11.22	0.313	-	-
Muscular thickness (μm)	99.17 ± 5.00 ^a^	103.53 ± 4.27 ^ab^	112.87 ± 7.80 ^b^	99.25 ± 6.83 ^a^	0.025	0.584	0.026
Crypt depth (μm)	60.23 ± 5.46	52.92 ± 6.09	47.28 ± 5.92	51.54 ± 4.11	0.112	-	-

Note: ^a–c^ Values in the same row with different letters indicate significant differences (*p* < 0.05), while that with the same letter or no letter superscripts indicate no significant differences (*p* > 0.05). AN: ANOVA, LT: linear trend, QT: quadratic trend.

## Data Availability

The data presented in this study are available in the main article.
